# Time-Dependent Changes in T1 during Fracture Healing in Juvenile Rats: A Quantitative MR Approach

**DOI:** 10.1371/journal.pone.0164284

**Published:** 2016-11-10

**Authors:** Katharina Baron, Bernhard Neumayer, Eva Amerstorfer, Eva Scheurer, Clemens Diwoky, Rudolf Stollberger, Hanna Sprenger, Annelie M. Weinberg

**Affiliations:** 1 Ludwig Boltzmann Institute for Clinical-Forensic Imaging (LBI CFI), Graz, Austria; 2 Department of Paediatric and Adolescent Surgery, Medical University of Graz, Graz, Austria; 3 Institute of Forensic Medicine, University of Basel – Health Department Basel, Basel, Switzerland; 4 Institute of Molecular Biosciences, University of Graz, Graz, Austria; 5 Institute of Medical Engineering, Graz University of Technology, Graz, Austria; 6 Department of Orthopedics and Orthopedic Surgery, Medical University of Graz, Graz, Austria; 7 BioTechMed-Graz, Graz, Austria; Indiana University Purdue University at Indianapolis, UNITED STATES

## Abstract

Quantitative magnetic resonance imaging (qMRI) offers several advantages in imaging and determination of soft tissue alterations when compared to qualitative imaging techniques. Although applications in brain and muscle tissues are well studied, its suitability to quantify relaxation times of intact and injured bone tissue, especially in children, is widely unknown. The objective observation of a fracture including its age determination can become of legal interest in cases of child abuse or maltreatment. Therefore, the aim of this study is the determination of time dependent changes in intact and corresponding injured bones in immature rats via qMRI, to provide the basis for an objective and radiation-free approach for fracture dating. Thirty-five MR scans of 7 Sprague-Dawley rats (male, 4 weeks old, 100 ± 5 g) were acquired on a 3T MRI scanner (TimTrio, Siemens AG, Erlangen, Germany) after the surgical infliction of an epiphyseal fracture in the tibia. The images were taken at days 1, 3, 7, 14, 28, 42 and 82 post-surgery. A proton density-weighted and a T1-weighted 3D FLASH sequence were acquired to calculate the longitudinal relaxation time T1 of the fractured region and the surrounding tissues. The calculation of T1 in intact and injured bone resulted in a quantitative observation of bone development in intact juvenile tibiae as well as the bone healing process in the injured tibiae. In both areas, T1 decreased over time. To evaluate the differences in T1 behaviour between the intact and injured bone, the relative T1 values (bone-fracture) were calculated, showing clear detectable alterations of T1 after fracture occurrence. These results indicate that qMRI has a high potential not only for clinically relevant applications to detect growth defects or developmental alterations in juvenile bones, but also for forensically relevant applications such as the dating of fractures in cases of child abuse or maltreatment.

## Introduction

The exact determination of the age of a fracture is a legally relevant question in cases where children show multiple fractures in different stages of bone healing and suspicion of maltreatment exists. However, the determination of a fracture’s age in young children is currently mainly based on the assessment of qualitative radiological data [1,2], resulting in an urgent need for objective, precise and quantitative methods for the analysis of fractures in young individuals.

The well-studied bone healing process starts with an inflammatory response followed by a primary soft callus formation, callus mineralisation and is completed with bone remodelling. During these general phases of fracture healing several anabolic as well as catabolic processes occur [3–6]. Furthermore, the juvenile bone itself is still in growth, showing a high amount of different physiological processes, since it has a highly regenerative ability compared to elderly bones [7]. However, detailed transitions of phases and changes in metabolic processes are not visible in radiographic imaging and thus the single stages may not be captured by using this method. Therefore, the assessment of a fracture may sometimes be challenging with standard radiographs due to a lack of sufficient image information.

Magnetic resonance imaging (MRI), on the other hand, provides a high soft tissue contrast and is potentially capable of showing more details in fracture repair. Moreover, MRI provides more information on the tissues surrounding the fractured area, including bone marrow, muscles, periosteum and fat [8–10].

Qualitative changes of signal intensity of 3D FLASH MR images are consistent with the presence of haematomas and changes during the healing process, confirmed by histological analysis of injured rat tibiae [11]. Since qualitative methods for the dating of fracture injuries are sometimes rather based on personal clinical experience than evidence, quantitative measures may become more suitable for children. Additionally, Baron et al described time dependent changes in quantitative MRI (qMRI) measurements of fractures in adult subjects as a precursor for a quantitative fracture dating approach. Therefore, MRI could become the more adequate method for the diagnosis and dating of a fracture [8,10].

Dating of fractures becomes especially important in cases of inflicted fractures in children. Therefore, the primary goal of this study is to systematically investigate alterations in intact and corresponding injured bones over time by using qMRI in immature Sprague-Dawley rats. This should provide the basis for an objective and radiation-free approach for fracture analysis in children.

## Materials and Methods

This work is based on the MRI examination of injured tibiae of 7 rats out of a cohort of 72 Sprague-Dawley rats (male, 4 weeks old, weight 100 ± 5 g) from a study published by Fischerauer et al. in 2011 [11]. The study was carried out in strict accordance with national and international guidelines and approved by the Committee on the Ethics of Animal Experiments of the Federal Ministry of Science, Research and Economy Austria (BMBWK-66.010/0054, BVGT/2006).

### Surgical procedure

All surgical proceedings were performed and described in [11]. Anaesthesia was induced by subcutaneous injections of Fentanyl^®^ (25 μg/kg), Dormitor^®^ (Medetomidine, 0.5 mg/kg) and Dormicum^®^ (Midazolam, 5 mg/kg). The left leg was shaved and sterilized using Betaisodona^®^. The articular surface of the proximal tibia was exposed by a parapatellar skin incision, followed by a subcutaneous dissection and an arthrotomy in line with the skin incision. An epiphyseal fracture was induced with an electric drill (Draimel^®^) thereby creating a cylindrical hole of the tibial head of 1.2 mm in diameter, which is running from the centre of the tibial plateau towards the tibial medullary cavity (fractured region) and passing through the tibial cortex. A Vicryle^®^ 5.0 suture was used for wound closure prior to antagonising the anaesthesia with an intraperitoneal injection of Annexate^®^ (Flumazenil, 0.5 mg/kg), Naloxon^®^ (1.2 mg/kg) and Antisedan^®^ (Atipamezol, 2.5 mg/kg) solution. Rats were monitored at all times until returning to full consciousness. For postoperative analgesia all rats received Rimadyl^®^ (Carprofen, 4–5 mg/kg/day, subcutaneously) during the first, and Novalgin^®^ (10 gtt. per 100 ml of drinking water) during the second week. During the period of the study all rats received food and water ad libitum [11].

### MRI examination and data analysis

The bone fractures of 7 rats (5 individuals at each point in time, two individuals were replaced due to premature death) were scanned at day 1, 3, 7, 14, 28, 42 and 82 after fracture initiation (Table 1), using a clinical 3T MRI scanner with 38 mT/m gradient strength (TimTrio, Siemens AG, Erlangen, Germany). All scans were acquired in coronal orientation. The animals were anesthetised at all times during the MRI examination in accordance with the aforementioned procedure (section *Surgical procedure*). The animals were placed in prone position with their knees centred on an 18 mm surface coil (Rapid Biomedical, Rimpar, Germany) in order to maintain a maximized signal-to-noise ratio (SNR) during the study.

**Table 1:** MR measurements after fracture initiation: x marks the scanning times of the individual rats.

Timepoint (daf)	Animal 1	Animal 2	Animal 3	Animal 4	Animal 5	Animal 6	Animal 7
**1**	x	X	x	x	x		
**3**	x	x	x	x	x		
**7**	x	x		x	x	x	
**14**	x	x		x		x	X
**28**	x	x		x		x	X
**42**	x	x		x		x	X
**82**	x	x		x		x	X

daf: days after fracture initiation

A PD-weighted 3D FLASH sequence (TE 2.95 ms, TR 100 ms, FA 5°, matrix 205 x 256, field of view 50 mm, slice thickness 0.72 mm) was acquired, followed by a T1-weighted 3D FLASH sequence (TE 2.95 ms, TR 8.09 ms, FA 30°, matrix 205 x 256, field of view 50 mm, slice thickness 0.72 mm), which was acquired 3 times in a row. These 3 measurements were averaged to obtain one T1-weighted image with increased SNR. Both, the PD-weighted and the T1-weighted image were used for the calculation of the longitudinal relaxation time T1.

A total of 35 MR scans were quantitatively evaluated twice, using a customized Matlab GUI (R2014a, MathWork Inc.).

**Fig 1:**
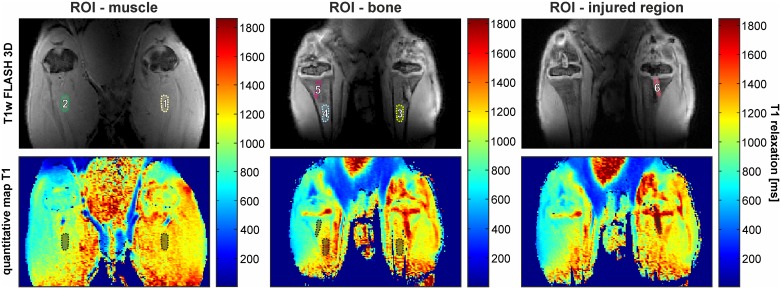
MRI examination of the tibial region of Sprague-Dawley rats. Top: T1w FLASH 3D images of the lower limbs, scanned in prone position. The ROIs are highlighted in dashed coloured lines to illustrate their approximate locations (locations of original ROIs may vary due to the slice thickness). The ROI of the muscle of the injured and intact leg are referred to as ROI 1 (light yellow) and ROI 2 (green), respectively. The ROI of the intact bone of the injured leg (light green) is denoted as ROI 3. ROI 4 (light blue) is located on the intact bone of the intact leg—mirroring ROI 3. The ROI of the intact bone of the intact leg, which mirrors the injured (fractured) region, is referred to as ROI 5 (pink). ROI 6 (red) highlights the injured region. Bottom: Equivalent quantitative T1 maps of the examined muscle regions (left), the examined bone regions (middle) and injured tibia region (right), scanned in prone position. The colour scale represents increasing relaxation time T1 (from blue to red). The ROIs are highlighted in black.

A single observer defined the regions of interest (ROI) by outlining cross sectional areas of the gastrocnemius muscle surrounding the fractured area (ROI 1), the corresponding muscle in the intact leg (ROI 2), the intact bone (diaphysis) of the tibia of the fractured (ROI 3) and intact (ROI 4) leg, the intact bone of the intact leg mirroring the fractured region of the injured leg (ROI 5), and each injured region (fracture; ROI 6) (see Fig 1). Due to subsequent acquisitions it was necessary to define slices that resembled closely the one used for the previous acquisition to ensure constant ROI determination. Therefore, ROIs of the muscles and intact bones were held constant over time in size and as similar as possible regarding their location and boundaries. The average ROI of muscle was 70 pixels and for the bones of the intact as well as the injured leg the ROI had an average pixel size of 40. The boundaries of ROIs of the injured region slightly varied on the one hand as a consequence of the growth of the individuals and following unavoidable adjustment of the orientation of the 3D slab along the bone, and on the other hand due to the healing process of the injured region. In each case the contamination of ROIs with fascia, subcutaneous and intermuscular fat, bone haematoma and major bone fat was avoided. These defined ROIs were then transferred to the quantitative maps to calculate the spatial median, which is determined as the median longitudinal relaxation time T1 of the defined ROI of one specific slide. The calculation is based on a method published by Hittmair et al. [12],using the equation proposed by Merwa et al. in 2009 [13]:

$$T1=\;-\;\frac{TR_{DCE}}{ln(\frac{S_{REF}\;sin\;\left(\theta_{DCE}\right)-S_{DCE}\;sin(\theta_{REF})}{S_{REF}sin\;\left(\theta_{DCE}\right)-S_{DCE}\;sin\left(\theta_{REF}\right)\;cos(\theta_{DCE})})}$$

where TR is the repetition time, S the signal intensity in the image, θ the flip angle and the subscripts REF and DCE denote the reference and dynamic scan, respectively.

All ROIs were chosen in a way that the individual T1 relaxation time per pixel was normally distributed within the ROIs. This was performed for all tissues with a standard deviation of around 60–80 ms within one ROI for muscle and 60–100 ms for bones. Standard deviation for T1 within one ROI of the injured region varied due to the vast amount of different substances as a consequence of the bone healing within the defective side.

### Statistics

The statistical analysis of all T1 relaxation times was performed using SPSS 22 (SPSS Chicago, IL). The intra-observer correlation coefficient (ICC) was calculated using a two-way random consistency ICC calculation between two repeated measurements of one observer of each, muscles (ROI 1), bones (ROI 3) and fracture (ROI 6).

Furthermore, this method was implemented to compare bone measurements between ROI 3 and ROI 4, as well as ROI 4 and 5 to determine whether the position of the ROI has an influence on T1 values in general.

Student´s t-tests were performed for each point in time between muscle measurements (ROIs 1 and 2) and between bones (ROIs 3 and 4; ROIs 4 and 5) separately (treating every point in time as a specific group), to indicate potential statistically significant differences between the ROIs independent from time.

The change of the T1 relaxation time of the gastrocnemius muscle (ROI 1) surrounding the fractured region, the intact bone of the injured leg (ROI 3) and the relative fracture values over time were analysed using one-way ANOVA prior to specific group comparisons using post hoc Tukey. In all three cases assumptions regarding normal distribution (Shapiro-Wilk test for small group sizes) and homogeneity of variance (Levene’s test) were made.

The Spearman’s rank correlation coefficient was calculated to measure the relation between changes in T1 relaxation times of the fracture gap over time. Furthermore, a Kruskal-Wallis H test was performed for values of the gastrocnemius muscle of the intact leg (ROI 2) and the fractured region (ROI 6), since heterogeneity of variances was confirmed with Levene’s test. A pairwise comparison requires a significant Kruskal-Wallis H-test and was performed only in the fractured region.

Potential outliers (definition: minor outlier 1.5x interquartile range (IQR), major outlier 2x 1.5x IQR) were calculated.

All results were visualised using box plots. Possible outliers were indicated in the box plots; however, they were included in the statistical analysis due to the small sample size.

## Results and Discussion

The aim of this study was to examine alterations in intact and corresponding injured bones over time by using qMRI in immature Sprague-Dawley rats.

All fractures showed a regular healing process during the course of the study and the formation of bone bridges within the epiphyseal lesion was confirmed with qualitative MRI [11]. The MR images showed good anatomical coverage despite the physical development of the animals.

### Intra-observer reliability

The intra-observer reliability, based on double measurements of the ROIs of the muscle (ROI 1), bone (ROI 3) and fractured region (ROI 6), was tested with the intra-observer correlation coefficient resulting in *r*_*I*_ = 0.941 (muscle; ROI 1), *r*_*I*_ = 0.851 (injured tibia; ROI 3) and *r*_*I*_ = 0.576 (fractured region, ROI 6) (p<0.05 for all measurements). Therefore, the measurements of all three anatomical regions were reproducible. The low value of the correlation coefficient of the fracture region may be explained by the different physiological processes and substances centred in particular in the injured region influencing the MRI signal.

### Quantitative MR muscle analysis

A qMRI analysis of the gastrocnemius muscles of the intact and the injured leg was implemented to provide reference values and to validate the quantitative approach of this study. There was no statistically significant difference between the muscle of the intact and injured legs (Student’s t-test for each point in time). These results indicate independency of the position of the ROI, when analysing muscles in an injured or intact leg. However, this requires that the analysed muscle in the injured leg is not directly connected with the injured region itself.

**Fig 2:**
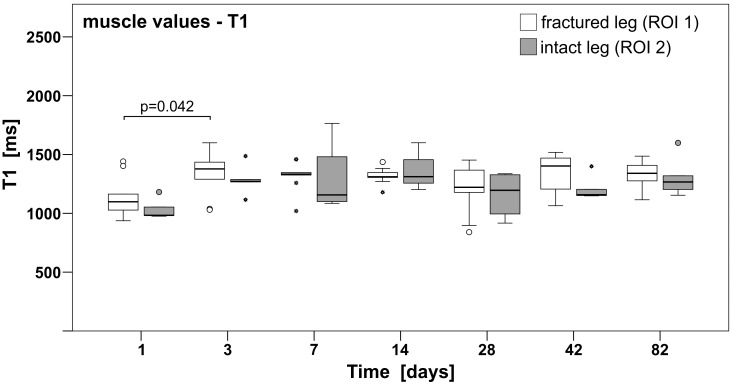
T1 relaxation of gastrocnemius muscles remained constant from day 3 on. Combined T1 values of the injured and intact gastrocnemius muscles. The overall median T1 values of the gastrocnemius muscle were 1202.45 ms (mean ± SD: 1264.34 ± 189.99 ms) for the intact and 1325.66 ms (mean ± SD: 1282.13 ± 168.50 ms) for the injured leg, respectively. Post hoc Tukey results are indicated (p = 0.042 between day 1 and 3 in the fractured leg). Horizontal bars indicate the median, the 25^th^ and 75^th^ percentiles, minor outliers are displayed with a circle; major outliers with a star.

The group median values of the gastrocnemius muscles for the intact as well as the injured leg are shown in Fig 2 and Table 2. The overall median (as well as means ± standard deviation (SD)) of T1 of the gastrocnemius of the intact and injured leg were 1202.45 ms (mean ± SD: 1264.34 ± 189.99 ms) and 1325.66 ms (mean ± SD: 1282.13 ± 168.50 ms), respectively, and remained constant over time from the third day onwards.

**Table 2:** Median (as well as mean ± standard deviation) of T1 (ms) over time for all measured muscle, bone and fracture values separated by days after fracture.

Muscle values	1^st^ day after fracture	3^rd^ day after fracture	7^th^ day after fracture	14^th^ day after fracture	28^th^ day after fracture	42^nd^ day after fracture	82^nd^ day after fracture
ROI 2 (intact leg)(overall: 1202.45 ms)	983.1 ms (1035.24 ± 87.41)	1271.15 ms (1285.57 ± 132.15)	1156.47 ms (1290.72 ± 319.79)	1311.50 ms (1356.25 ± 170.50)	1195.71 ms (1161.23 ± 202.60)	1158.00 ms (1212.01 ± 107.01)	1266.51 ms (1308.22 ± 174.03)
ROI 1 (injured leg; repeated measurements) (overall: 1325.66 ms)	1089.25 ms (1130.72 ±173,69)	1377.55 ms (1349.74 ± 194.19)	1333.77 ms (1318.79 ± 129.37)	1310.71 ms (1317.31 ± 71.35)	1221.45 ms (1210.27 ± 201.09)	1402.49 ms (1330.24 ± 165.42)	1340.26 ms (1329.84 ± 114.58)
**Bone values**							
ROI 4(intact leg)	1090.91 ms (1100.66 ± 129.16)	1486.07 ms (1475.77 ± 86.53)	1303.08 ms (1307.35 ± 220.43)	1221.13 ms (1248.10 ± 156.40)	1220.87 ms (1230.21 ± 153.23)	855.22 ms (986.21 ± 319.41)	764.60 ms (807.50 ± 205.72)
ROI 5 (intact leg)	1072.8 ms (1033.92 ± 74.94)	1279.69 ms (1302.15 ± 164.02)	1171.45 ms (1205.15 ± 179.93)	1143.48 ms (1198.18 ± 122.25)	1161.36 ms (1184.78 ± 165.42)	987.91 ms (985.38 ± 121.10	863.25 ms (172.62 ± 172.62)
ROI 3 (injured leg; repeated measurements)	1204.07 ms (1254.09 ± 178.39)	1585.33 ms (1635.65 ± 173.49)	1317.69 ms (1351.22 ± 200.53)	1425.92 ms (1411.30 ± 153.34)	1398.22 ms (1385.96 ± 147.79)	1067.41 ms (1091.67 ± 204.03)	862.99 ms (822.16 ± 159.85)
**Fracture values**							
ROI 6 (injured leg; repeated measurements)	1572.7 ms (1739.63 ± 459.2)	1589.21 ms (1582.20 ± 171.9)	1017.59 ms (1099.70 ± 238.02)	1100.6 ms (1189.08 ± 141.3)	1250.82 ms (1201.76 ± 144.95)	1083.6 ms (1044.20 ± 147.04)	929.9 ms (911.86 ± 107.03)
**Relative fracture values**							
ROI 3 –ROI 6 (injured leg; Bone—Fracture; repeated measurements)	-436.47 ms (-485.5 ± 354.5)	96.63 ms (53.3 ± 233.5)	314.40 ms (270.36 ± 321.1)	207.4 ms (222.2 ± 225.5)	145.04 ms (172.3 ± 196.7)	-45.41 ms (47.4 ± 293.0)	-88.60 ms (-89.7 ± 150.3)

The comparison of this outcome with T1 values of human test subjects as well as rodents known from literature ([10,14–17]; Table 3) showed consistent results, which indicates a successful approach validation.

**Table 3:** Comparison of T1 of musculature in lower extremities (human, mouse and rat).

	Morrow et al. [14]	Jordan et al. [15]	Gold et al. [16]	Baron et al. [10]	Stanisz et al [17]	This study (incl. day 1)
	human	human	human	human	mouse	rat
**T1 (ms)**	~1000.0–1500.0	1255.9 ± 57.9	1420 ± 38.1	1280 ± 216	1412 ± 13	1264.34 ± 189.99 ms (intact leg) and 1282.13 ± 168.50 ms (injured leg)

However, ANOVA showed significant differences of T1 (p<0.02) and post hoc Tukey revealed significantly different T1 values between day 1 and 3 (p = 0.04), since T1 values of muscle of the injured leg (ROI 1) were below average on day 1, compared to median values of later measurements (for further details see Table A in S1 File).

The gastrocnemius muscle of the intact leg (ROI 2) showed no statistically significant difference (Kruskal-Wallis H test) over time; however, T1 on day 1 was below the average as well (Fig 2).

Nonsteroidal anti-inflammatory drugs (NSAIDs) are described as having the potential of considerably impacting physiological processes, although, the actual impact of NSAID is estimated differently in literature [18–20]. The authors assume that the lower T1 values on day 1 probably stem from side-effects due to the administration of the NSAID Rimadyl^®^ (Carprofen) within the first post-operative days. Since inflammatory responses in fractures peak within the initial 24 hours [4], the impact of NSAIDs is supposed to be highest during this timeframe. The influence of the surgery itself and the following recovery on T1 may be neglected since a comparable effect was observed in the intact leg. Also the impact of temporary changes in the water and food intake is negligible as all rats received water and food ad libitum during the study and no dietary refusals were observed during the first days after surgery. The average weight of all rats increased constantly during the course of the study. Altogether, the authors infer that a systemic effect due to medication is most likely. However, a specific study regarding changes in relaxation time due to different drug administration may be considered to evaluate the low T1 values observed on the first day.

### Quantitative MR bone analysis

T1 values of the intact tibiae were chosen as reference values since it was assumed that T1 values of the fracture gap return to values of healthy bone over time. Therefore, T1 relaxation time of the tibial diaphysis—including bone marrow—of the injured (ROI 3) and the intact leg (ROI 4) were measured with the chosen quantitative approach. A two-way random consistency ICC calculation (between diaphysis of injured and intact leg) was performed to determine whether physiological processes of the injury influence the T1 values of ROI 3, which resulted in *r*_*I*_ = 0.859 with p<0.001 between ROI 3 and ROI 4. Furthermore, no statistically significant differences were observed between these ROIs using the Student’s t-test for each point in time separately.

However, in order to compare the injured region with intact bone regions it was necessary to further test if the position of the ROI on the tibia (dia- or metaphysis; ROI 4 and ROI 5) influences T1. Hence, a ROI at the metaphysis of the intact leg (ROI 5)–mirror-inverted to ROI 6 of the injured leg—was chosen (see Fig 1). The two-way random consistency ICC between ROI 4 and ROI 5 was *r*_*I*_ = 0.649 with p = 0.02. A comparison between the mirror-inverted metaphysis (ROI 5) and the diaphysis (ROI 4) of the intact tibia did not reveal significant differences by analysing each point in time separately (Student´s t-test) except for day three.

Both results indicate that T1 of the bone is independent of the location of the ROI, since dia- and metaphysis reveal similar T1 values. This justifies the comparison between intact diaphysis and injured metaphysis of the injured leg to observe the development of bone healing in the fractured region. Hence, this is also in line with clinical practice, where imaging is performed only in the injured region.

**Fig 3:**
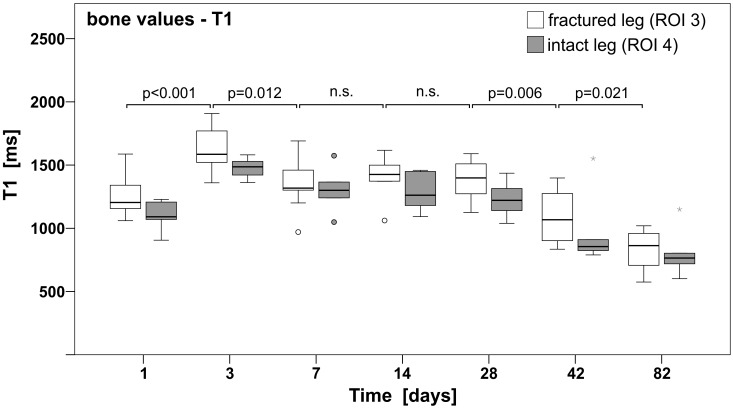
T1 relaxation of the tibial bones from fractured and intact legs showed a decrease from childhood towards adulthood [21,22]. T1 measurements of the injured and intact tibial bones over time. Post hoc Tukey results are indicated (n.s. = not significant). Horizontal bars indicate the median, the 25^th^ and 75^th^ percentiles. The major outliers are displayed with a star; minor outliers with a circle.

Additionally, the temporal progress of T1 of the intact bone (ROI 3) was investigated. A decrease of T1 relaxation times during the developmental stages from childhood and adolescence to adulthood (juvenile/child 21–32 days old, peripubertal/adolescent 33–55 days old, late puberty/adolescent 56–70 days old, [21,22]) was observed (Fig 3, Table 2), and ANOVA revealed a statistical significance between the values over time (p<0.001). The post hoc Tukey test showed significantly different results between several days (especially between day 3 and the other days); for further details see Table B in S1 File. To exclude influences on T1 values due to inconsistencies in longitudinal growth of the bones, the length of the tibia of both legs was measured at each time point in all individuals. No differences in the length of the tibial bone between intact and injured limbs were observed.

Results of this study show the temporal progress of T1 in the developmental stages in rats. It is known from literature that during childhood and adolescence, in rats as well as in humans, the skeleton grows in length and diameter and constantly remodels during development (reviewed in [22]),([23,24] cited in [25]). In humans bone mass increases throughout puberty to reach its maximum peak in adult bone within the second life decade ([23,24] cited in [25]). Simultaneously, changes within the bone marrow occur, recognisable by a diminution of red (cellular) bone marrow towards yellow (stromal) marrow, which causes an increase of fat within these regions [26–28]. It is assumed by the authors that the mentioned alterations in the composition of the bone marrow in combination with changes in cell composition and cytokine related cell differentiation as described by [29], may also provoke the change of T1 relaxation time during bone development in rats.

### Quantitative analysis of the injured areas via MRI

**Fig 4:**
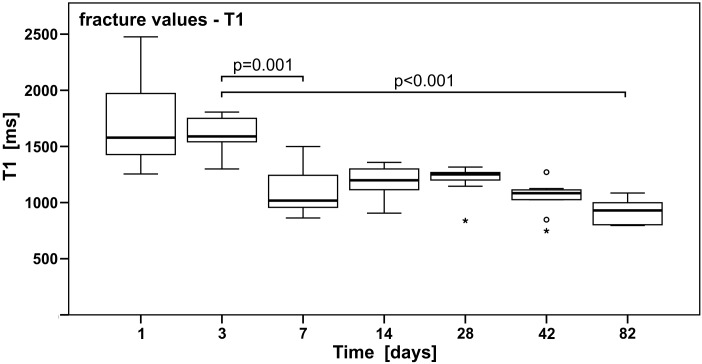
Processes of fracture healing are reflected by a constant decline in T1 relaxation of the tibial fracture gap. T1 measurements of the fracture gap. Results of a pairwise comparison following Kruskal-Wallis H test are indicated. Horizontal bars indicate the median, the 25^th^ and 75^th^ percentiles. The major outliers are displayed with a star; minor outliers with a circle.

The chosen fracture model is based on a surgical model of the epiphysis and metaphysis, with a concomitant lesion of the physis (which was not the main interest of this study). This pattern resembles a typical Aitken III or Salter-Harris IV fracture which is routinely present in injured children with fractures in the physeal region. This gave the authors the opportunity to examine injured bone regions near the epiphyseal plates while these plates are still open. The tibial fracture gap of immature rats was investigated via qMRI to assess, if and to what extent physiological changes in the fracture gap influence T1 behaviour over time. Fig 4 and Table 2 show median T1 values of the fractured area over time, grouped according to the days after fracture infliction.

Spearman´s rank order correlation between T1 values of the fractured areas and time showed a strong negative and significant correlation (r_*s*_ = -0.75; p<0.001). The Kruskal-Wallis H test for independent samples showed significant results (p<0.001). Pairwise comparison between T1 of the fractured region on days 1 and 3 showed a p-value of 1; however, days 1 and 3 were significantly different to days 7, 42, and 82 with p-values <0.001 (for further details see Table C in S1 File).

The beginning of the healing process on day 1 was defined by a comparatively high variation in data. This might be explained by the different extent of haematoma formation, soft tissue injuries and inflammatory responses between animals, which was also proposed by Baron et al. for adult human individuals [10]. In general, a negative correlation of T1 was observed over time. Baron et al. [10] published a similar trend of T1 in healing fractures of adult human subjects. They proposed that an increase of paramagnetic substances like methaemoglobin within the haematoma cavity may cause a decrease in T1. From literature it is known that hyper-intense signals in T1-weighted images (short T1) can indicate increased amounts of proteins, laminar necrosis, and presence of paramagnetic substances [10,30,31].

NSAIDs may cause prolonged bone healing in different rodent studies [32] and therefore have the potential to induce temporary changes in T1 relaxation times, as described for muscle and bone values in this study. Therefore, it deems possible that the starting point of T1 relaxation time might be even higher on day 1 than in the herein presented results of the fracture gap.

However, high turnover rates during bone recovery after the inflammatory response (increase in skeletal blood flow) in the fractured region cause a temporarily higher rate of haematopoietic (red) marrow, which is then replaced by yellow marrow from day 14 onwards (partially observable in the histological images of [11]). Moreover, since the drilled fracture leads through the growth plate a slight bias in all results may be present due to a possible material transfer from the growth plate to the fracture gap. According to Fischerauer et al., bone debris was observed merely during the first 7 days, which then formed bony trabeculae seen on day 14. On day 28 it was observed that the trabeculae turned into bone bridges, which indicates advanced bone healing compared to models where the debris was removed [11,32].

Therefore, the authors consider two opposing effects that influence T1 relaxation time of the injured region. Firstly, NSAID could provoke temporarily lower T1 values than expected on day 1 and secondly, even though NSAID may prolong the healing process itself, the bone debris appears to promote faster healing of the injured region.

### Relative fracture MR values

**Fig 5:**
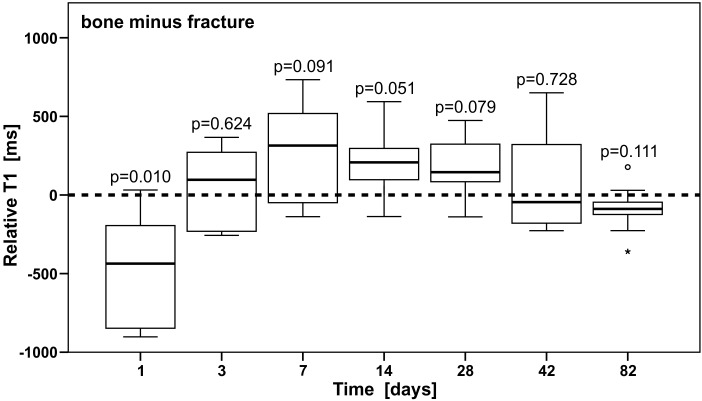
T1 relaxation of the fractured region assimilates to T1 of the intact bone over time. T1 values of the fracture gap in relation to the intact bone by subtracting fracture values from bone values. Statistical results (Student’s t-tests) are indicated (p-value). Horizontal bars indicate the median, the 25^th^ and 75^th^ percentiles. The major outlier is displayed with a star; the minor outlier with a circle.

Fig 5 and Table 2 show changes in T1 of the fracture gap related to intact bone values (by subtraction) over time, in order to illustrate that T1 values of the injured bone returned to T1 values of healthy bone (relative T1 of approx. zero) during the healing process.

Separate one-sample Student’s t-tests between relative fracture values and zero were conducted by treating each point in time separately. The fracture in comparison with the bone had significantly different T1 values on day 1 (the relative value was significantly different to zero, p = 0.010). On days 7, 14 and 28 the relative T1 values exceeded zero but were not reaching statistical significance (p = 0.091, 0.051 and 0.079, respectively).

The normal distribution and variance homogeneity were tested with Shapiro-Wilk and Levene’s test. The overall comparison of the relative values with ANOVA showed significantly different T1 (p<0.001), which allowed to implement the post hoc Tukey analysis for a group wise comparison. The relative values on days 3, 7, 14, 28 and 42 were not statistically significant to each other (for further details see Table D in S1 File).

The author’s infer the positive values (days 7, 14, 28), i.e. shorter T1 values for the injured area, to be linked to a transient increase of bone density during bone remodelling. However, confirming this hypothesis unambiguously will require additional histological analysis and the acquisition of further MR parameters.

## Conclusion

This study aims to systematically investigate time-resolved changes in T1 relaxation times of a surgical lesion of the epiphysis and metaphysis in juvenile Sprague-Dawley rats, to determine whether or not qMRI is suitable to detect the bone healing process in juvenile subjects. Within the course of this study three main outcomes were achieved.

Firstly, it was demonstrated that the bone development and therefore bone remodelling can be observed via qMRI and described by changes of T1 relaxation times over time. It is hypothesized that this effect is induced by the diminution of red bone marrow towards yellow bone marrow and further varying growth factors during the development of the animals.

Secondly, the applicability of qMRI to analyse bone fracture healing in juvenile Sprague-Dawley rats was successfully determined. Similar to the bone values itself, a decrease of T1 in the fractured region could be observed over time. It is supposed that the influx of paramagnetic substances into the haematoma cavity of the fracture shortens T1. However, additional quantitative MR sequences and histology of corresponding regions will be required to determine the different stages of bone healing and the age of a fracture based on quantitative methods. These supplementary methods would allow for deepening the knowledge in influencing factors of the various substances that are distributed in the fracture gap. The relative fracture T1 values, i.e. bone T1 minus fracture T1, indicated differing T1 behaviour of intact bone and fractured areas. This may enable the differentiation between intact and healing bone in a future fracture dating approach when transferring these results to children.

Thirdly, relatively low T1 values of muscle, bone and the fracture were noticed on day 1 after the surgery. The potential side effects of NSAIDs on bone healing processes, which are frequently described in literature, may explain the low T1 relaxation times of muscle, bone and the fractured area in early phases of bone healing. However, a detailed investigation of the effects of NSAID or other factors on changes in T1 during bone development and reconstruction were not part of this study.

Altogether, the gained results clearly underline the high potential for the applications of a radiation-free method for detecting growth defects or developmental alterations of juvenile bones. However, the impact of these findings in a human approach has to be further clarified. Further studies pointing in this direction are highly recommended. Beyond that, it deems possible that experts in the field of forensic age estimation of fractures could benefit from the results of this study since the herein gained knowledge on specific tissue relaxation parameters could provide the basis for an objective, radiation-free and quantitative approach to determine a fracture’s age.

## Supporting Information

S1 File Supplemental statistical results. (DOCX)
