# Supplementary material for: Time-Dependent Changes in T1 during Fracture Healing in Juvenile Rats: A Quantitative MR Approach
Source: PLoS One. 2016 Nov 10;11(11):e0164284. doi: 10.1371/journal.pone.0164284 (PMC5104481; doi:10.1371/journal.pone.0164284)
Supplement: S1 File — (DOCX) [file pone.0164284.s001.docx]

| **S1 File: Supplemental statistical results**  **Table A: Post hoc Tukey analysis of intact muscle of the injured leg (ROI 1) over time.** | | | | | | | |
| --- | --- | --- | --- | --- | --- | --- | --- |
| (A) Time (days after fracture) | (B) Time (days after fracture) | Mean difference (A-B) | Standard error | p-value | | 95 % Confidence interval | |
|  |  |  |  |  |  | lower level | Upper level |
| 1 | 3 | -219,02800^*^ | 70,26084 | | **,**042 | -433,3629 | -4,6931 |
|  | 7 | -188,07233 | 72,18615 | | ,143 | -408,2805 | 32,1358 |
|  | 14 | -186,59122 | 72,18615 | | ,149 | -406,7994 | 33,6169 |
|  | 28 | -79,55500 | 70,26084 | | ,916 | -293,8899 | 134,7799 |
|  | 42 | -199,51900 | 70,26084 | | ,084 | -413,8539 | 14,8159 |
|  | 82 | -199,11900 | 72,18615 | | ,102 | -419,3272 | 21,0892 |
| 3 | 1 | 219,02800^*^ | 70,26084 | | ,042 | 4,6931 | 433,3629 |
|  | 7 | 30,95567 | 72,18615 | | ,999 | -189,2525 | 251,1638 |
|  | 14 | 32,43678 | 72,18615 | | ,999 | -187,7714 | 252,6449 |
|  | 28 | 139,47300 | 70,26084 | | ,435 | -74,8619 | 353,8079 |
|  | 42 | 19,50900 | 70,26084 | | 1,000 | -194,8259 | 233,8439 |
|  | 82 | 19,90900 | 72,18615 | | 1,000 | -200,2992 | 240,1172 |
| 7 | 1 | 188,07233 | 72,18615 | | ,143 | -32,1358 | 408,2805 |
|  | 3 | -30,95567 | 72,18615 | | ,999 | -251,1638 | 189,2525 |
|  | 14 | 1,48111 | 74,06143 | | 1,000 | -224,4477 | 227,4099 |
|  | 28 | 108,51733 | 72,18615 | | ,742 | -111,6908 | 328,7255 |
|  | 42 | -11,44667 | 72,18615 | | 1,000 | -231,6548 | 208,7615 |
|  | 82 | -11,04667 | 74,06143 | | 1,000 | -236,9755 | 214,8821 |
| 14 | 1 | 186,59122 | 72,18615 | | ,149 | -33,6169 | 406,7994 |
|  | 3 | -32,43678 | 72,18615 | | ,999 | -252,6449 | 187,7714 |
|  | 7 | -1,48111 | 74,06143 | | 1,000 | -227,4099 | 224,4477 |
|  | 28 | 107,03622 | 72,18615 | | ,754 | -113,1719 | 327,2444 |
|  | 42 | -12,92778 | 72,18615 | | 1,000 | -233,1359 | 207,2804 |
|  | 82 | -12,52778 | 74,06143 | | 1,000 | -238,4566 | 213,4010 |
| 28 | 1 | 79,55500 | 70,26084 | | ,916 | -134,7799 | 293,8899 |
|  | 3 | -139,47300 | 70,26084 | | ,435 | -353,8079 | 74,8619 |
|  | 7 | -108,51733 | 72,18615 | | ,742 | -328,7255 | 111,6908 |
|  | 14 | -107,03622 | 72,18615 | | ,754 | -327,2444 | 113,1719 |
|  | 42 | -119,96400 | 70,26084 | | ,614 | -334,2989 | 94,3709 |
|  | 82 | -119,56400 | 72,18615 | | ,647 | -339,7722 | 100,6442 |
| 42 | 1 | 199,51900 | 70,26084 | | ,084 | -14,8159 | 413,8539 |
|  | 3 | -19,50900 | 70,26084 | | 1,000 | -233,8439 | 194,8259 |
|  | 7 | 11,44667 | 72,18615 | | 1,000 | -208,7615 | 231,6548 |
|  | 14 | 12,92778 | 72,18615 | | 1,000 | -207,2804 | 233,1359 |
|  | 28 | 119,96400 | 70,26084 | | ,614 | -94,3709 | 334,2989 |
|  | 82 | ,40000 | 72,18615 | | 1,000 | -219,8082 | 220,6082 |
| 82 | 1 | 199,11900 | 72,18615 | | ,102 | -21,0892 | 419,3272 |
|  | 3 | -19,90900 | 72,18615 | | 1,000 | -240,1172 | 200,2992 |
|  | 7 | 11,04667 | 74,06143 | | 1,000 | -214,8821 | 236,9755 |
|  | 14 | 12,52778 | 74,06143 | | 1,000 | -213,4010 | 238,4566 |
|  | 28 | 119,56400 | 72,18615 | | ,647 | -100,6442 | 339,7722 |
|  | 42 | -,40000 | 72,18615 | | 1,000 | -220,6082 | 219,8082 |

*. Significance: p<0.05, highlighted in yellow

|  |
| --- |
|  |

| **Table B: Post hoc Tukey analysis of intact bone values of the injured leg (ROI 3) over time.** | | | | | | | |
| --- | --- | --- | --- | --- | --- | --- | --- |
| (A) Time (days after fracture) | (B) Time (days after fracture) | Mean difference (A-B) | Standard error | p-value | | 95 % Confidence interval | |
|  |  |  |  |  |  | lower level | Upper level |
| 1 | 3 | -381,55100^*^ | 77,81044 | | ,000 | -618,9164 | -144,1856 |
|  | 7 | -97,12767 | 79,94263 | | ,886 | -340,9974 | 146,7421 |
|  | 14 | -157,20656 | 79,94263 | | ,446 | -401,0763 | 86,6632 |
|  | 28 | -131,86600 | 77,81044 | | ,622 | -369,2314 | 105,4994 |
|  | 42 | 162,42900 | 77,81044 | | ,373 | -74,9364 | 399,7944 |
|  | 82 | 431,93678^*^ | 79,94263 | | ,000 | 188,0670 | 675,8065 |
| 3 | 1 | 381,55100^*^ | 77,81044 | | ,000 | 144,1856 | 618,9164 |
|  | 7 | 284,42333^*^ | 79,94263 | | ,012 | 40,5536 | 528,2931 |
|  | 14 | 224,34444 | 79,94263 | | ,091 | -19,5253 | 468,2142 |
|  | 28 | 249,68500^*^ | 77,81044 | | ,033 | 12,3196 | 487,0504 |
|  | 42 | 543,98000^*^ | 77,81044 | | ,000 | 306,6146 | 781,3454 |
|  | 82 | 813,48778^*^ | 79,94263 | | ,000 | 569,6180 | 1057,3575 |
| 7 | 1 | 97,12767 | 79,94263 | | ,886 | -146,7421 | 340,9974 |
|  | 3 | -284,42333^*^ | 79,94263 | | ,012 | -528,2931 | -40,5536 |
|  | 14 | -60,07889 | 82,01940 | | ,990 | -310,2840 | 190,1262 |
|  | 28 | -34,73833 | 79,94263 | | ,999 | -278,6081 | 209,1314 |
|  | 42 | 259,55667^*^ | 79,94263 | | ,030 | 15,6869 | 503,4264 |
|  | 82 | 529,06444^*^ | 82,01940 | | ,000 | 278,8594 | 779,2695 |
| 14 | 1 | 157,20656 | 79,94263 | | ,446 | -86,6632 | 401,0763 |
|  | 3 | -224,34444 | 79,94263 | | ,091 | -468,2142 | 19,5253 |
|  | 7 | 60,07889 | 82,01940 | | ,990 | -190,1262 | 310,2840 |
|  | 28 | 25,34056 | 79,94263 | | 1,000 | -218,5292 | 269,2103 |
|  | 42 | 319,63556^*^ | 79,94263 | | ,003 | 75,7658 | 563,5053 |
|  | 82 | 589,14333^*^ | 82,01940 | | ,000 | 338,9383 | 839,3484 |
| 28 | 1 | 131,86600 | 77,81044 | | ,622 | -105,4994 | 369,2314 |
|  | 3 | -249,68500^*^ | 77,81044 | | ,033 | -487,0504 | -12,3196 |
|  | 7 | 34,73833 | 79,94263 | | ,999 | -209,1314 | 278,6081 |
|  | 14 | -25,34056 | 79,94263 | | 1,000 | -269,2103 | 218,5292 |
|  | 42 | 294,29500^*^ | 77,81044 | | ,006 | 56,9296 | 531,6604 |
|  | 82 | 563,80278^*^ | 79,94263 | | ,000 | 319,9330 | 807,6725 |
| 42 | 1 | -162,42900 | 77,81044 | | ,373 | -399,7944 | 74,9364 |
|  | 3 | -543,98000^*^ | 77,81044 | | ,000 | -781,3454 | -306,6146 |
|  | 7 | -259,55667^*^ | 79,94263 | | ,030 | -503,4264 | -15,6869 |
|  | 14 | -319,63556^*^ | 79,94263 | | ,003 | -563,5053 | -75,7658 |
|  | 28 | -294,29500^*^ | 77,81044 | | ,006 | -531,6604 | -56,9296 |
|  | 82 | 269,50778^*^ | 79,94263 | | ,021 | 25,6380 | 513,3775 |
| 82 | 1 | -431,93678^*^ | 79,94263 | | ,000 | -675,8065 | -188,0670 |
|  | 3 | -813,48778^*^ | 79,94263 | | ,000 | -1057,3575 | -569,6180 |
|  | 7 | -529,06444^*^ | 82,01940 | | ,000 | -779,2695 | -278,8594 |
|  | 14 | -589,14333^*^ | 82,01940 | | ,000 | -839,3484 | -338,9383 |
|  | 28 | -563,80278^*^ | 79,94263 | | ,000 | -807,6725 | -319,9330 |
|  | 42 | -269,50778^*^ | 79,94263 | | ,021 | -513,3775 | -25,6380 |
| *. Significance: p<0.05, highlighted in yellow | | | | | | | |

| **Table C: Pairwise comparison of the injured region (ROI 6) over time.**  Each row tests the null hypothesis whether the distribution between Group 1 and Group 2 is equal. Asymptotic significance (two tailed testing) is presented. Significance level: p<0.05. | | | | | | | | |  |
| --- | --- | --- | --- | --- | --- | --- | --- | --- | --- |
| Group 1 (days after fracture) | Group 2 (days after fracture) | Test statistics | Standard error |  | | | |  |  |
|  |  |  |  | p-value | | | Adjusted sig. |  |  |
| 1  42  82  42  82  7  7 | 3 | 33.500 | 8.456 | | 1 | .002 | | | |
|  | 1 | 44.222 | 8.687 | | .000 | .000 | | | |
|  | 3 | 33.500 | 8.456 | | .000 | .002 | | | |
|  | 3 | 44.222 | 8.687 | | .000 | .000 | | | |
|  | 1 | 30.875 | 8.969 | | .001 | .012 | | | |
|  | 3 | 30.875 | 8.969 | | .001 | .012 | | | |

| **Table D: Post hoc Tukey analysis of relative fracture values over time.** | | | | | | |
| --- | --- | --- | --- | --- | --- | --- |
| (A) Time (days after fracture) | (B) Time (days after fracture) | Mean difference (A-B) | Standard error | p-value | 95 % Confidence interval | |
|  |  |  |  |  | lower level | Upper level |
| 1 | 3 | -538,99000^*^ | 117,65111 | ,000 | -898,3145 | -179,6655 |
|  | 7 | -755,90125^*^ | 124,78784 | ,000 | -1137,0224 | -374,7801 |
|  | 14 | -707,76111^*^ | 120,87502 | ,000 | -1076,9319 | -338,5903 |
|  | 28 | -657,85667^*^ | 120,87502 | ,000 | -1027,0275 | -288,6859 |
|  | 42 | -533,00600^*^ | 117,65111 | ,001 | -892,3305 | -173,6815 |
|  | 82 | -395,83556^*^ | 120,87502 | ,028 | -765,0063 | -26,6648 |
| 3 | 1 | 538,99000^*^ | 117,65111 | ,000 | 179,6655 | 898,3145 |
|  | 7 | -216,91125 | 124,78784 | ,594 | -598,0324 | 164,2099 |
|  | 14 | -168,77111 | 120,87502 | ,802 | -537,9419 | 200,3997 |
|  | 28 | -118,86667 | 120,87502 | ,956 | -488,0375 | 250,3041 |
|  | 42 | 5,98400 | 117,65111 | 1,000 | -353,3405 | 365,3085 |
|  | 82 | 143,15444 | 120,87502 | ,897 | -226,0163 | 512,3252 |
| 7 | 1 | 755,90125^*^ | 124,78784 | ,000 | 374,7801 | 1137,0224 |
|  | 3 | 216,91125 | 124,78784 | ,594 | -164,2099 | 598,0324 |
|  | 14 | 48,14014 | 127,83189 | 1,000 | -342,2780 | 438,5583 |
|  | 28 | 98,04458 | 127,83189 | ,987 | -292,3736 | 488,4627 |
|  | 42 | 222,89525 | 124,78784 | ,563 | -158,2259 | 604,0164 |
|  | 82 | 360,06569 | 127,83189 | ,089 | -30,3525 | 750,4838 |
| 14 | 1 | 707,76111^*^ | 120,87502 | ,000 | 338,5903 | 1076,9319 |
|  | 3 | 168,77111 | 120,87502 | ,802 | -200,3997 | 537,9419 |
|  | 7 | -48,14014 | 127,83189 | 1,000 | -438,5583 | 342,2780 |
|  | 28 | 49,90444 | 124,01515 | 1,000 | -328,8568 | 428,6657 |
|  | 42 | 174,75511 | 120,87502 | ,775 | -194,4157 | 543,9259 |
|  | 82 | 311,92556 | 124,01515 | ,173 | -66,8357 | 690,6868 |
| 28 | 1 | 657,85667^*^ | 120,87502 | ,000 | 288,6859 | 1027,0275 |
|  | 3 | 118,86667 | 120,87502 | ,956 | -250,3041 | 488,0375 |
|  | 7 | -98,04458 | 127,83189 | ,987 | -488,4627 | 292,3736 |
|  | 14 | -49,90444 | 124,01515 | 1,000 | -428,6657 | 328,8568 |
|  | 42 | 124,85067 | 120,87502 | ,944 | -244,3201 | 494,0215 |
|  | 82 | 262,02111 | 124,01515 | ,359 | -116,7401 | 640,7823 |
| 42 | 1 | 533,00600^*^ | 117,65111 | ,001 | 173,6815 | 892,3305 |
|  | 3 | -5,98400 | 117,65111 | 1,000 | -365,3085 | 353,3405 |
|  | 7 | -222,89525 | 124,78784 | ,563 | -604,0164 | 158,2259 |
|  | 14 | -174,75511 | 120,87502 | ,775 | -543,9259 | 194,4157 |
|  | 28 | -124,85067 | 120,87502 | ,944 | -494,0215 | 244,3201 |
|  | 82 | 137,17044 | 120,87502 | ,915 | -232,0003 | 506,3412 |
| 82 | 1 | 395,83556^*^ | 120,87502 | ,028 | 26,6648 | 765,0063 |
|  | 3 | -143,15444 | 120,87502 | ,897 | -512,3252 | 226,0163 |
|  | 7 | -360,06569 | 127,83189 | ,089 | -750,4838 | 30,3525 |
|  | 14 | -311,92556 | 124,01515 | ,173 | -690,6868 | 66,8357 |
|  | 28 | -262,02111 | 124,01515 | ,359 | -640,7823 | 116,7401 |
|  | 42 | -137,17044 | 120,87502 | ,915 | -506,3412 | 232,0003 |
| *. Significance: p<0.05, highlighted in yellow | | | | | | |
